# Case Report: Successful Use of Biliary Stent for Iatrogenic Esophageal Perforation Following Balloon Dilation in a 7-Month-Old Infant

**DOI:** 10.3389/fped.2020.545760

**Published:** 2020-10-28

**Authors:** Meng-Chuan Liu, Yao-Sheng Wang, Yao-Jong Yang, Fu-Ping Lai

**Affiliations:** ^1^Department of Pediatrics, National Cheng Kung University Hospital, Tainan, Taiwan; ^2^Department of Internal Medicine, National Cheng Kung University Hospital, Tainan, Taiwan; ^3^Institute of Clinical Medicine, Medical College, National Cheng Kung University, Tainan, Taiwan

**Keywords:** endoscopic balloon dilation, esophageal perforation, endoscopic therapy, esophageal stent, pediatrics—infants, biliary stent

## Abstract

Esophageal perforation is a rare but critical emergency that requires early detection and prompt management. In the pediatric population, iatrogenic injury is the most common etiology of esophageal perforation, and the majority of cases come from stricture dilation. Treatment options include medical management, endoscopic therapy, and surgery. Usually, conservative treatment is appropriate in most carefully selected patients, especially in the setting of early diagnosis and with the absence of severe sepsis. A surgical approach is reserved for a large tear with mediastinum contamination, or clinical deterioration after unsuccessful conservative management. With the advancement of the endoscopy technique, endoscopy therapy using esophageal stents is an available choice for adult populations who have a complicated protracted healing course or comorbidities precluding surgical attempts. However, this procedure is seldom implemented in children, especially in young infants, owing to unavailable equipment and experts. We report our successful use of a fully-covered self-expandable metal biliary stent in managing esophageal perforation in a seven-month-old infant. In light of this encouraging achievement, this model can be applied to more children who have the same problem.

## Introduction

Esophageal atresia (EA) is one of the common congenital esophageal malformations in children. EA with and without tracheoesophageal fistula (TEF) is classified into five types: EA without TEF (type A), EA with proximal TEF (type B), EA with distal TEF (type C), EA with both proximal and distal TEF (type D), and TEF without EA (type E, H-type TEF). Type C is the most common form of EA. Standard surgical intervention has been TEF ligation and esophageal end-to-end anastomosis. In the case of H-type TEF, bronchoscopy combined with endoscopy management has also been attempted (1). Although the survival of patients with EA has significantly improved since Haight and Towsley (2) reported their first primary repair in 1944, complications after EA repair remain a continuing challenge. Esophageal anastomotic stricture is the major early complication after the surgery, accounting for up to two-thirds of the patients (3). Endoscopic dilatation with balloon or savary dilators is a suggested procedure to manage post-operative anastomotic stricture with a satisfactory outcome of 79.7% (4). However, repeated esophageal dilation is needed for recurrent and refractory anastomotic stricture (5), which may increase the risk of esophageal perforation.

Esophageal perforation is a serious and potentially life-threatening condition that requires early detection and prompt management. Treatment options include medical management, endoscopic therapy, and surgery. Conservative treatment is appropriate in most carefully selected patients, especially in the setting of early diagnosis and absence of severe sepsis, while a surgical approach is reserved for a large tear with mediastinum contamination and sepsis, or clinical deterioration after unsuccessful conservative management (6–8). In patients with esophageal perforation who have a poor healing process or advanced comorbidities that preclude surgical approach, endoscopic esophageal stenting has been widely applied to adult populations (9). However, endoscopic esophageal stenting in pediatric populations is uncommon due to the limitations of available instruments and techniques. We report our successful use of a fully covered self-expandable metal biliary stent in managing iatrogenic esophageal perforation in a 7-month-old infant.

## Case Report

A female infant weighing 2,942 g was born at 39^+4^ weeks gestation by vaginal delivery. She was found to have a tracheoesophageal fistula and esophageal atresia (TEF/EA) soon after birth. The right aortic arch with an aberrant left subclavian artery was diagnosed in a complete assessment of the VACTERL association. She temporarily received laparoscopic esophageal banding and gastrostomy one day after birth. The thoracoscopic repair of TEF/EA was conducted when she was 2 months old. However, esophageal anastomotic stricture ensued 2 months later. She cannot tolerate oral feeding smoothly and received serial endoscopic balloon dilation at an interval of 3 weeks. At 7 months old, she received the 6^th^ session of esophageal dilation with a balloon diameter of 9 mm. Five hours after the procedure, the patient became severely tachypneic with labored breathing. Chest auscultation revealed diminished breathing sounds over the left lung field. The respiratory rate was 55 per minute, heart rate was 195 per minute, blood pressure was 103/72 mmHg, and SpO_2_ was 98% under a simple oxygen face mask. A chest X-ray disclosed left pneumothorax and pneumomediastinum with a mediastinal shift. Emergent pigtail catheter insertion and endotracheal tube intubation were performed. Chest computed tomography revealed a suspected esophageal mucosal injury, pneumopericardium, pneumomediastinum, and left pneumothorax. Under the diagnosis of esophageal perforation, she received conservative treatment, including avoidance of oral intake, nasogastric tube decompression, intravenous broad-spectrum antibiotics, and partial parenteral nutrition. The clinical course was complicated by fever, but the patient soon became hemodynamically stable.

**Figure 1:**
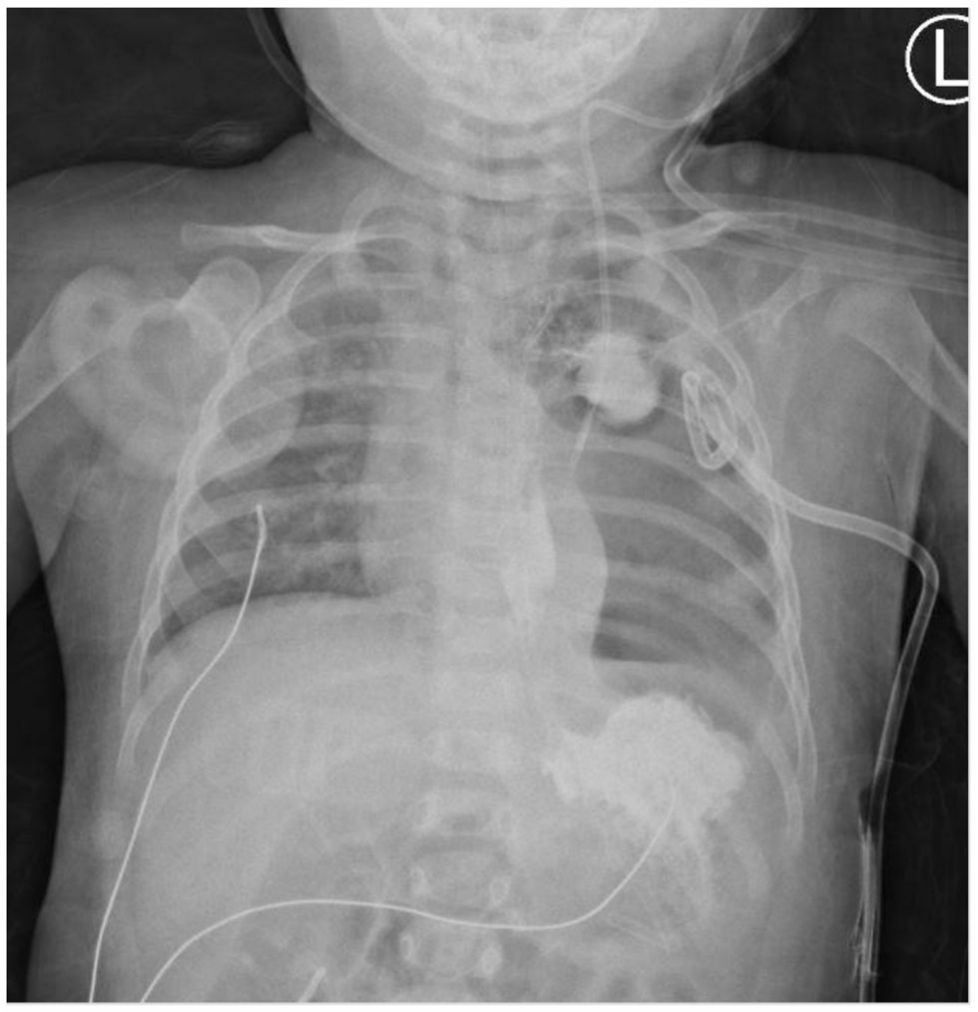
Esophageal perforation with the contrast leak contained in left focal para-mediastinal space.

**Figure 2:**
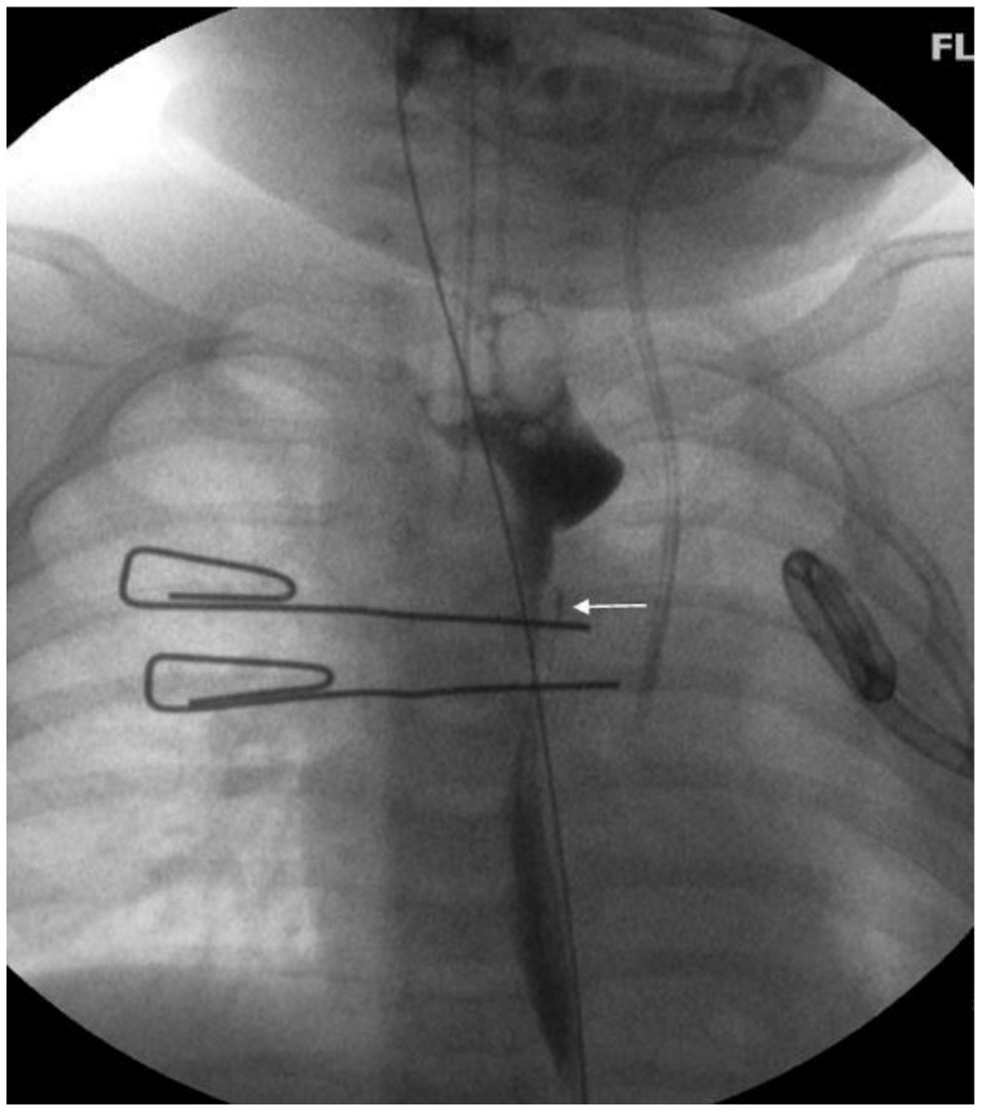
Esophageal stricture and a trivial contrast leak (arrow).

**Figure 3:**
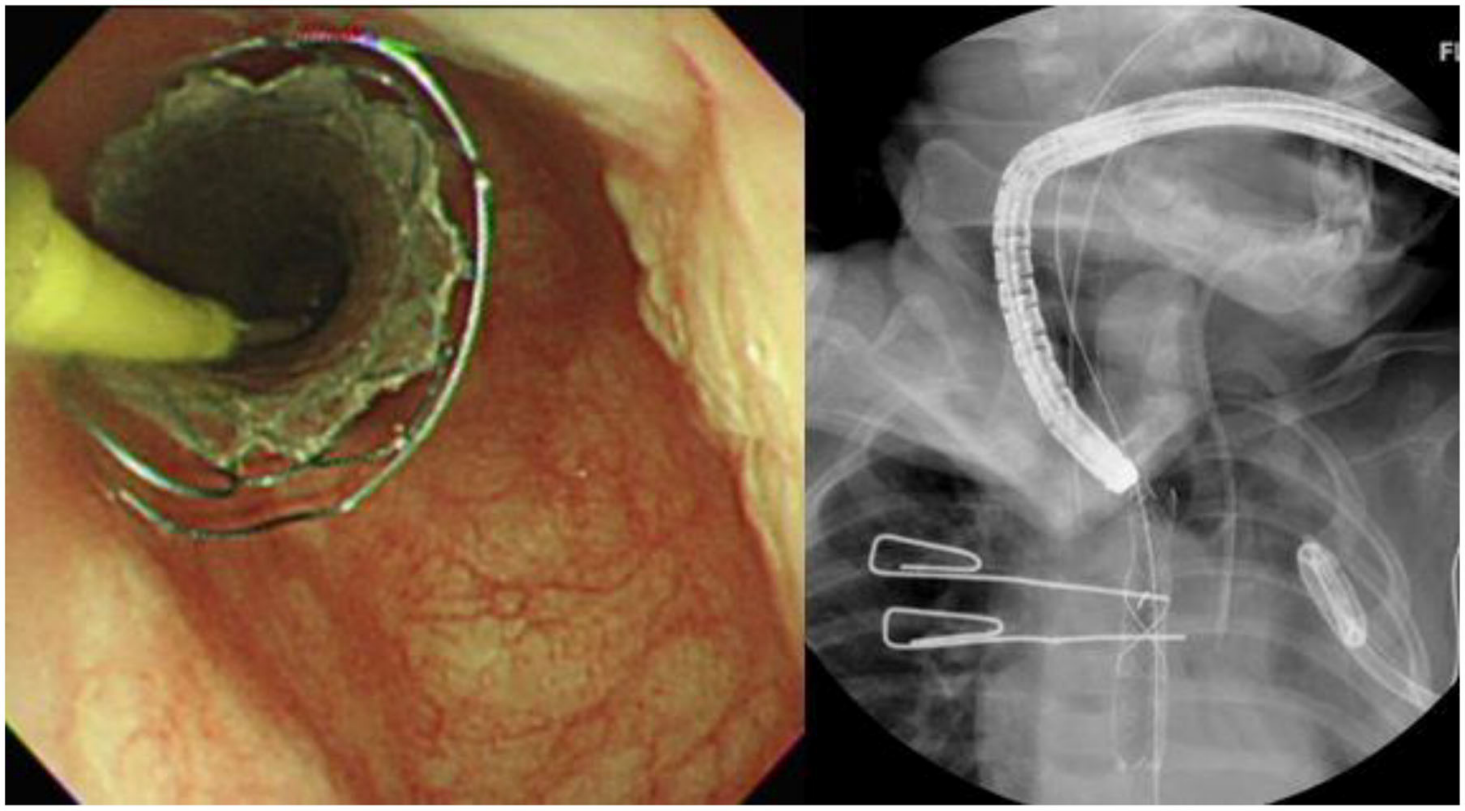
Placement of a biliary stent in the esophagus under endoscopic and fluoroscopic guidance.

**Figure 4:**
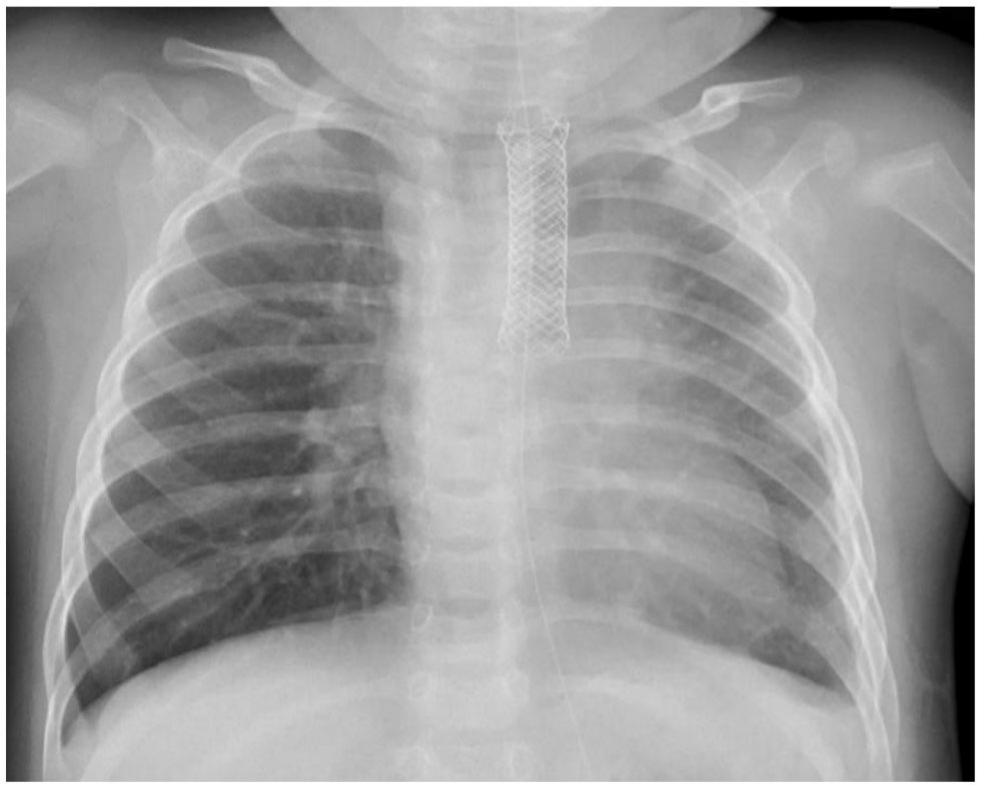
Replacement of a new biliary stent.

She was extubated and started on NG-tube feeding 4 days after the event. However, milk-like fluid drained out from the pigtail catheter after advancing the feeding amount. A chest X-ray after being orally fed with water-soluble contrast revealed contrast leakage with local accumulation over the left lung (Figure 1). Fasting was resumed, along with total parenteral nutrition support. However, follow-up chest X-rays showed progressing left-sided pleural effusion. Persistent esophageal leakage after a 14-day conservative treatment was impressed. She received an esophageal stent placement under endoscopic and fluoroscopic guidance. A stricture in the mid-esophagus and a trivial contrast leak was noticed under the concurrent esophagogram (Figure 2). A fully covered self-expandable metal biliary stent (M-intraductal biliary stent, the middle portion diameter was 8 mm, and the diameter at the both ends was 10 mm) was applied across the esophageal stricture because small-diameter esophageal stents for children were unavailable (Figure 3). Two weeks later, the upward migration of the esophageal stent was noticed. Endoscopic assessment following the removal of the dislodged stent disclosed a lesser degree of the stricture, but there was a small oval wall defect over it. A new fully covered self-expanding metal biliary stent was placed (WallFlex Biliary Stent, diameter 10 mm, length 40 mm; Figure 4). One month later, an esophagogram demonstrated an improvement in the stricture with no more contrast leakage. The esophageal stent was removed. The patient tolerated oral intake smoothly during a 4-month follow-up period.

## Discussion

Esophageal perforation is a potentially life-threatening clinical emergency with a high mortality and morbidity rate. In the pediatric population, iatrogenic injury is the most common etiology of esophageal perforation, and the majority comes from stricture dilation (10). Early diagnosis and intensive medical and/or surgical management promise good outcomes, and successful non-operative management require careful patient selection. The criteria for non-operative management of esophageal perforations had been established, consisting of perforations that are early diagnosed, not in neoplastic tissue or in the abdominal cavity, and the symptoms and signs of septicemia should be absent (6). Since iatrogenic injuries are often diagnosed more quickly and are associated with less extraluminal contamination, most of them are appropriate for non-operative attempts.

However, in patients who fail to respond to conservative management or have a refractory leak, endoscopic esophageal stents provide an alternative with increasing clinical application. Rollins and Barnhart (11) reported three children with esophageal perforations that were managed successfully with covered nitinol esophageal stents, after the perforations failed to close with non-operative management. The stents were introduced 13–29 days after perforation for 3–4 weeks. All of them sealed the perforation successfully. Manfredi et al. (12) also reported a success rate of 80% for closure of esophageal perforations with stent therapy after dilation. A systematic review demonstrated a success rate of 76% using self-expanding esophageal stents for managing esophageal perforation in children (13). The optimal amount of time to leave the stent in place for esophageal perforation remains unclear, and a repeat esophagogram is mandated to document that the perforation has sealed. According to the European Society for Gastrointestinal Endoscopy Guidelines (14), a duration of 6–8 weeks of stent insertion was recommended for benign esophageal stricture and esophageal perforation. In our case, complete healing of perforation and relief of stricture was achieved after 7 weeks of stenting, which conformed with previous studies.

Adverse events after esophageal stent placement should be monitored regularly. Migration is one of the most common complications, occurring in up to 56% of the pediatric population (15) and up to 75% of adult patients (16). The risk of migration is associated with the stent location, the presence of strictures, the use of plastic rather than metal stents, and the use of fully covered stents (17). In our case, although we used a fully covered self-expandable metal biliary stent with a middle waist and a cross-wire structure for preventing early migration, upward migration still developed 2 weeks later. Using a fully covered stent could be a factor. However, considering that the uncovered section of partially covered stents may allow tissue embedment, and this may make stent removal difficult and traumatic, we replaced it with a new, fully covered self-expanding metal biliary stent once again.

Hemorrhage due to arterioesophageal fistula after esophageal stent placement has been reported in patients with aberrant subclavian artery (18, 19). Though our case had right aortic arch with an aberrant left subclavian artery, since esophagectomy was challenging in our case, and hemorrhage due to vascular erosion is infrequent in the previous studies (20), the esophageal stent was still applied.

Another challenge of esophageal stenting in the pediatric population is that the size of current commercially available esophageal stents may be inappropriate for small children. Using tracheobronchial stents or self-expanding metal biliary stents as an alternative has been reported in narrow esophageal indications (11, 21–23). Custom dynamic stents, which allow food to pass between the stent and esophageal wall, have also been used (24). Our case demonstrates that biliary stents can be used safely in cases of esophageal perforation in infants. Nevertheless, the development of esophageal stents that are flexible, resist migration, and are available in smaller sizes for the pediatric population desires exploitation in the future.

## Data Availability Statement

The raw data supporting the conclusions of this article will be made available by the authors, without undue reservation, to any qualified researcher.

## Ethics Statement

We obtained informed written consent from the patient's parent authorizing publication of clinical case and images.

## Author Contributions

M-CL is a resident on the Pediatric Gastroenterology team who drafted the majority of the case report. Y-SW is the gastroenterologist who conducted the endoscopic esophageal stenting procedures. Y-JY is the attending on the Pediatric Gastroenterology team who provided treatment and follow-up for the patient throughout the course of the illness. He also contributed to the editing of the case report and the final manuscript. F-PL is the attending on the Pediatric Gastroenterology team. She contributed to the editing of the case report and the final manuscript. All authors approved the final manuscript as submitted and agree to be accountable for all aspects of the work.

## Conflict of Interest

The authors declare that the research was conducted in the absence of any commercial or financial relationships that could be construed as a potential conflict of interest.
